# Calendar

**DOI:** 10.1155/S1463924680000363

**Published:** 1980


### Calendar

**Editor's Note:**
*Organisers of conferences, seminars etc. should send details for inclusion in this calendar as soon as the relevant information is available and not later than three months before the event.*

**High resolution spectroscopy**
April 13-15, Bristol
*Dr G Duxbury, School of Chemistry, University of Bristol, Cantocks' Close, Bristol, UK.*

**Laboratory safety**
April 16, London
*A.H. Thomas, National Institute for Biological Standards and Control, Holly Hill, Hampstead, London NW3, UK.*

**Contract research for the pharmaceutical and chemical industries**
April 21-22, Winchester
*Conference Manager, Entropy Scientific, Winters Road, Shirrell Heath, Hants, UK.*

**Advanced polarographic techniques**
April 21-23, Loughbrough
*Miss J M Brown, Dept of Chemistry, Loughbrough University of Technology, Loughbrough, Leics LE11 3TU, UK.*

**10th course on protein fractionation**
April 21-25, London
*The Secretary, Division of Biochemistry, North East London Polytechnic, Romford Road, London E15, UK.*

**2nd International Congress on Phosphorous Compounds**
April 21 – 25, Boston
*IMPHOS, 8 rue de Pentievre, 75008, Paris, France.*

**Labware '80**
April 22-24, London
*Labware Promotions, 28 Worple Road, London SW19 4EE, UK.*

**Newer techniques of analysis**
April 23, Birmingham
*Miss P E Hutchinson, Analytical Division, Chemical Society, Burlington House, London W1, UK.*

**Chemical information services symposium**
April 23, Manchester
*Dr P N K Riley, Chemistry Department, Manchester Polytechnic, Chester Street, Manchester M1, UK.*

**International Workshop on Trace Element Analytical Chemistry**
April 27-29, Neuherberg, West Germany
*Dr P Schramel, Gesellschaft fur Strahlen and Umweltforschung, Physikalisch-Technische Abteilung, Ingolstadter Landstrasse 1, D-8042 Neuherberg, FRG.*

**New techniques in the clinical laboratory**
April 28-29, Munich
*Robert S First Inc, 19a Avenue Marnix, 1050 Brussels, Belgium*

**Programming microprocessors for industrial measurement and control**
April 29-30, London
*Mrs R G Keiller, Sira Institute Ltd, South Hill, Chislehurst, Kent BR7 5EH, UK.*

**Analytica '80**
April 29 - May 2, Munich
*ECL (Exhibition Agencies) Ltd., 11 Manchester Square, London W1*

**Phirama 80 – Scientific and technical information exhibition**
May 6-9, Marseilles
*Foire de Marseilles, Service Phirama, Parc Chanot, 13008 Marseilles, France.*

**Radioimmunoassay – an intensive course**
May 12-16, London
*The Secretary, Division of Biochemistry, North East London Polytechnic, Romford Road, London EJ1, UK.*

**Particle size analysis and quality control for pharmaceuticals**
May 21, Sunderland
*Dr N A Orr, School of Pharmacy and Biology, Sunderland Polytechnic, Chester Road, Sunderland SR1, UK.*

**Analytical chemistry of pollutants**
May 28-30, Dortmund, FRG
*Dr J Wendenburg, Gesellschaft Deutscher Chemiker, PO Box 900440, D-6000 Frankfurt am Main 90, FRG*

**High speed automatic analysis**
May 30, Glasgow
*D. G. Porter, Laboratory of the Government Chemist, Cornwall House, Stamford St, London SE1 9NQ*

**Quantitative mass spectrometry in life sciences**
June 10-13, Gent, Belgium
*Prof A de Lenheer, Laboratoria voor Medische Biochemie en voor Klinische Analyse, De Pintelaan 135, B-9000 Gent, Belgium.*

**Analysis of non-metals in metals**
June 10-13, West Berlin
*Conference Office, Gesellschaft Deutscher Chemiker, Herrn Dr J Wendenburg, Postfach 90 04 40, D6000 Frankfurt/Main 90, West Germany.*

**Mass spectrometry in biochemistry, medicine and environmental research**
June 16-18, Milan
*Dr Alberto Frigerio, Instituto di Richerche Farmacologiche, 'Mario Negri', Via Eritrea, 62, 20157 Milan, Italy.*

**Eurochem '80**
June 23-27, Birmingham
*Andrew Dedman, Clapp & Poliak Europe Ltd, 232 Acton Lane, London W4 5DL, UK.*

**Electrophoretic techniques – Chemical Society Residential School**
June 30-July 4, Sussex
*Miss L Hart, Education Dept, Chemical Society, Burlington House, London W1, UK.*

**Swansea summer school of Automatic Chemical Analysis**
July 6-11, Swansea
*Dr J. Betteridge, University College of Swansea, Singleton Park, Swansea, Wales.*

**SAC 80**
July 20-26, Lancaster
*The Secretary, Analytical Division, Chemical Society, Burlington House, London W1V 0BN, UK.*

**Patenting strategies**
September 8-10, Manchester
*Miss L Hart, The Chemical Society, Burlington House, London W1, UK.*

**Laboratory '80**
September 9-11, London
*Curtis Steadman, 34-36 High Street, Saffron Walden, Essex.*

**3rd World Filtration Congress**
September 13-17, Philadelphia
*Filtration Society, Shippensburg, Philadelphia, USA.*

**New techniques in analytical instrumentation**
September 17-18, Amsterdam
*Robert S First Inc, 707 Westchester Ave, White Plains, New York 10604, USA.*

**Interkama 80 – 8th International Congress and Exhibition for Instrumentation and Automation**
October 9-15, Dusseldorf, West Germany
*Arbeitsgemeinscharft INTERKAMA, Postfach 700969, D-6000 Frankfurt/M 70, FRG.*

**1981**

**Euroanalysis IV**
August 23-28, Helsinki, Finland
*Association of Finnish Chemical Societies, Executive Secretary, Pohj, Hesperiankatu 3B10, SF-oo260 Helsinki 26, Finland*

